# eDNA-based assessment of microbial dynamics in response to persistent contaminants and degraded water quality in a hypereutrophic urban reservoir

**DOI:** 10.1007/s10661-026-15785-1

**Published:** 2026-08-22

**Authors:** Juliana S. M. Pimentel, Maria Clara V. M. Starling, Camila C. Amorim, Valquíria F. L. Viana, Alessandra S. Martins, Daniel A. S. Rodrigues, Luciana P. M. Brandão, Nadieh de Jonge, Niels O. G. Jørgensen, Louise Schlüter, Matt Simcik, Jeppe Lund Nielsen

**Affiliations:** 1 Intelligent Systems of Environmental Monitoring and Remediation (SIMOA), and Research Group On Environmental Applications of Advanced Oxidation Processes (GruPOA), Department of Sanitary and Environmental Engineering, Federal University of Minas Gerais, Av. Presidente Antônio Carlos 6627, Belo Horizonte, MG 31270-901 Brazil; 2 Department of Plant and Environmental Sciences, University of Copenhagen, Copenhagen, Denmark; 3 Department of Chemistry and Bioscience, Aalborg University, Aalborg, Denmark; 4 DHI, Environment and Toxicology, Hørsholm, Denmark; 5 School of Public Health, University of Minnesota, Minneapolis, USA; 6 Department of Chemistry, Federal University of Viçosa, Av. Peter Henry Rolfs, S/N, Viçosa, MG 36570-900 Brazil

**Keywords:** Environmental DNA, PFAS, Microbial community, Tropical reservoirs, Urban freshwater ecosystems, Metabarcoding

## Abstract

Urban freshwater ecosystems are increasingly exposed to mixtures of pollutants, including per- and polyfluoroalkyl substances (PFAS). However, their ecological effects remain poorly understood, especially in tropical regions with limited sanitation infrastructure. This study investigated PFAS contamination, water quality, and microbial community structure in Pampulha Lake, a tropical urban reservoir in southeastern Brazil. Spatiotemporal patterns in prokaryotic and eukaryotic diversity were assessed over a year (2022–2023) using environmental DNA metabarcoding of 16S and 18S rRNA genes by integrating taxonomic data with PFAS concentrations and water quality. Physicochemical and multivariate analyses revealed strong seasonal and spatial heterogeneity, influenced by eutrophication, hydrological variability, and pollution gradients. Among the seven PFAS detected above quantification limits, PFBS and PFOA were the most common. Prokaryotic communities showed significant correlations with PFAS, especially with sulfonated forms, while eukaryotic communities were more stable. Diversity patterns highlighted microbial community sensitivity to environmental stress. Overall, results indicate that PFAS influenced microbial assemblages, supporting their inclusion in monitoring frameworks, and eDNA was proved effective for assessing ecological risks in tropical urban reservoirs.

**Supplementary information:**

The online version contains supplementary material available at https://doi.org/10.1007/s10661-026-15785-1.

## Introduction

Freshwater ecosystems are increasingly exposed to complex mixtures of environmental contaminants, yet their ecological consequences remain poorly understood. Traditional ecotoxicological approaches typically focus on species-specific endpoints using vertebrate models, which are time-consuming and often difficult to extrapolate to community-level processes. In response, molecular-based monitoring tools such as environmental DNA (eDNA) have emerged as powerful, non-invasive alternatives for biodiversity assessment and ecological impact evaluation (Barnes & Turner, 2016; Ficetola et al., 2008).

eDNA refers to genetic material obtained directly from environmental matrices, including free DNA, whole cells, or entire microorganisms. Over the past decade, eDNA metabarcoding has become widely applied in terrestrial, marine, and freshwater environments due to its sensitivity and scalability. In aquatic ecosystems, this approach enables detection of rare or cryptic taxa while minimizing disturbance and was proven effective in reconstructing microbial and phytoplankton communities and their responses to environmental gradients (Lv et al., 2023; Thomsen & Willerslev, 2015; Valentini et al., 2016). The use of taxon-specific markers, such as the 16S rRNA gene for prokaryotes and 18S rRNA gene for eukaryotes, facilitates the identification of key taxa and their putative ecological roles relevant to ecosystem processes (Ray & Umapathy, 2022).

Urban lakes and reservoirs are particularly vulnerable to anthropogenic stressors, including nutrient enrichment, untreated sewage and industrial wastewater inputs, and the accumulation of persistent organic pollutants (POPs) such as per- and polyfluoroalkyl substances (PFAS). PFAS are highly stable compounds widely used in industrial and consumer products and are now ubiquitous in aquatic ecosystems due to their persistence (Podder et al., 2021). Even at low concentrations, PFAS have been shown to disrupt microbial assemblages, primary producers, and trophic interactions (Brunn et al., 2023). Recent studies have reported associations between PFAS exposure and shift in phytoplankton, zooplankton, and benthic communities (Dominique et al., 2025; Li et al., 2024), although responses remain inconsistent across taxa and ecosystems.

Despite growing concern, few studies have examined the effects of PFAS on microbial communities using eDNA-based approaches, particularly in tropical and urban freshwater systems that are both understudied and highly impacted (Xu et al., 2022). This study addresses this knowledge gap by investigating correlations between PFAS concentrations, physicochemical parameters, and microbial community structure in Pampulha Lake, a hypereutrophic urban reservoir in southeastern Brazil. We applied eDNA metabarcoding of the 16S rRNA- and 18S rRNA genes to characterize prokaryotic and eukaryotic assemblages across spatial and seasonal gradients. This study provides new insights into the ecological impacts of PFAS by integrating molecular diversity data with chemical and environmental parameters, and highlights the potential of eDNA as a tool for risk assessment and biomonitoring in tropical urban freshwater ecosystems.

## Materials and methods

### Study area

Pampulha Lake is an artificial reservoir located within the Velhas watershed in Belo Horizonte, Minas Gerais (MG), southeastern Brazil. The catchment area is completely urbanized and heavily impacted, with a total area of 2.7 km^2^, a perimeter of 21 km, and a maximum depth of 16 m. The lake receives inflow from eight direct tributaries, with the Ressaca and Sarandi streams supplying nearly 87% of the total water volume (Figueredo et al., 2016). Pampulha is a warm, monomictic, and eutrophic tropical reservoir, exhibiting thermal stratification in the rainy season (October to March) and vertical mixing during the dry season (April to September) (Brandão et al., 2016). The reservoir is characterized as hypereutrophic and algal blooms are common in the site (Figueredo et al., 2016). Cyanobacteria is generally the most dominant taxonomic group in the lake most of the year, represented mainly by the species *Cylindrospermopsis raciborskii* (Nostocales), *Planktothrix agardhii*, *P. isothrix* (Oscillatoriales), and *Microcystis aeruginosa* (Chroococcales). Other groups also occur, such as Chlorophyta, Bacillariophyta, and Euglenophyta, that are present but contribute less to the total biomass (Barçante et al., 2020). This is the first study with high-throughput next-generation sequencing (NGS) of bacterial and archaeal communities in Pampulha Lake.

### Sampling design

**Fig. 1:**
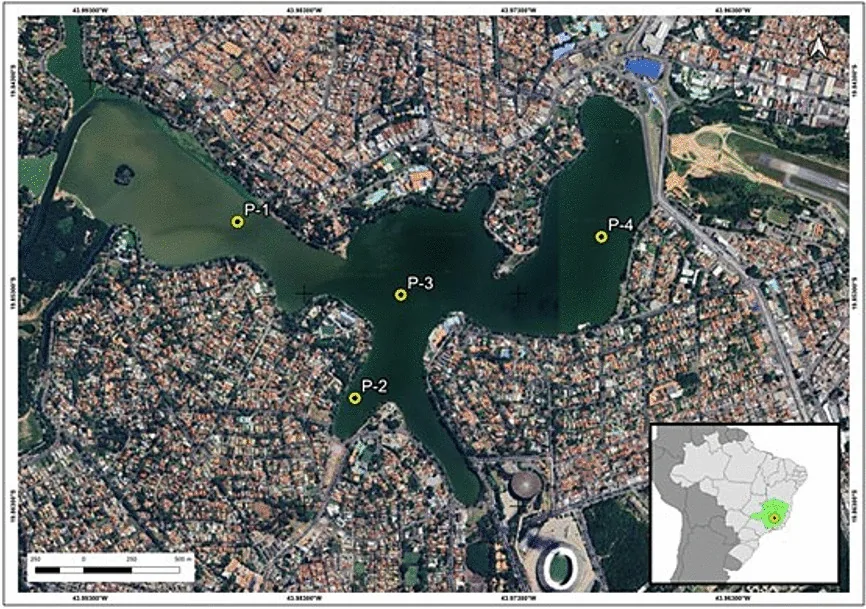
Sampling points in Pampulha Lake where water quality parameters were measured in situ and surface water samples were collected for environmental DNA (eDNA) and PFAS analysis

Surface water samples (collected at 30-cm depth) were obtained from four sites across Pampulha Lake: P1, P2, P3, and P4 (Fig. 1). Site P1 is located downstream from a surface water treatment unit (ETAF), which treats water from the Ressaca and Sarandi tributaries, both of which receive untreated sewage. A solid retention barrier downstream of P1 reduces suspended solids and coarse debris. These infrastructure elements influence physicochemical conditions at P1–P3. Site P4 is situated near the dam in the deepest part of the reservoir. Eight sampling campaigns were carried out between March 2022 and February 2023 (Table 1) encompassing both rainy and dry seasons. Analyses included eDNA profiling, PFAS quantification, and water quality parameters in duplicates.

**Table 1:** Sampling scheme and parameters analyzed in surface water from Pampulha Lake, MG, Brazil

Year	2022							2023
Season	Rainy	Dry				Rainy		
Parameter	Mar	Apr	May	Aug	Sep	Nov	Dec	Feb
PFAS	x	x	x	x	x	x	x	x
eDNA (16S and 18S)	x	x	x	x	x	x	x	x
Physicochemical parameters*	x	x	x	x	x	x	x	x

^*^Physicochemical parameters: Secchi depth, turbidity, pH, dissolved oxygen (DO), temperature, conductivity, ORP, TAL PC, chlorophyll-*α* (Chl α) and fluorescent dissolved organic matter (fDOM)

### Physicochemical analyses

Water quality parameters such as turbidity (Formazin Nephelometric Units; FNU), pH, DO (mg·L^−1^), water temperature (°C), electrical conductivity (EC, µS·cm^−1^), oxidation–reduction potential (ORP), total algae-phycocyanin (TAL PC), Chl-α (µg·L^−1^), and fluorescent dissolved organic matter (fDOM) were measured in situ using a EXO2-YSI® multiparameter probe. Average precipitation data corresponding to the sampling period were gathered from the Brazilian National Institute of Meteorology (INMET) records.

### Sample preparation and PFAS analysis

Water samples (250 mL) were filtered through 0.7-µm glass fiber filters (GF/F Whatman), and pH was adjusted to 6.0–7.0 using either formic acid or ammonium hydroxide. Extraction followed the US EPA Draft Method 1633 (EPA, 2021). Samples were spiked with 83 µL of extracted internal standard (EIS) and subjected to solid-phase extraction (SPE) using cartridges preconditioned with 15 mL 1% methanolic NH_4_OH and 5 mL 0.3 M formic acid. Cartridges were washed, vacuum-dried, and eluted with 1% NH_4_OH in MeOH followed by 25 µL of glacial acetic acid. Quality control included blanks (ultrapure water spiked with NIS), low-level ongoing precision and recovery (LLOPR), and OPR samples per batch. Final extracts were kept at − 4 °C prior to analysis. Quantification of PFAS was performed by high-performance liquid chromatography (HPLC, Agilent 1200 Series) coupled with tandem mass spectrometry using a triple quadrupole instrument (Sciex API 4000 LC–MS/MS). A detailed description of the methodology is available elsewhere (Starling et al., 2024).

### DNA extraction and quality control

Following sampling, 1–2 L of lake water samples were filtered through 0.22-µm MCE membranes. Filters were stored at − 20 °C prior to DNA extraction using the FastDNA Spin Kit for Soil (MP Biomedicals) according to the manufacturer’s protocol. DNA purity was assessed using the A260/A280 absorbance ratio using a NanoDrop UV–Vis spectrophotometer (Thermo Fisher Scientific). DNA integrity was verified by 0.8% agarose gel electrophoresis. Concentrations were measured using the Qubit™ 1 × dsDNA HS Assay Kit on a Qubit 4 fluorometer (Invitrogen, USA).

### Amplicon sequencing

The microbial and microeukaryotic communities were analyzed using 16S rRNA and 18S rRNA gene sequencing, respectively, using well-described primer sets targeting the V4 region of the 16S rRNA gene (Caporaso et al., 2011), and the V1–V2 region of the 18S rRNA gene (Blaxter et al., 1998; Sinniger et al., 2016). Samples were prepared for Nanopore sequencing in accordance with Grammatiki et al. (2025) with minor modifications. Briefly, for the amplicon PCR procedure, 30 cycles of PCR were used instead of 35, and 100 fmol of the respective 16S rRNA and 18S rRNA gene amplicon from the same sample was pooled prior to PCR barcoding and library preparation. The final library was sequenced on a R9.4.1 flowcell using an Oxford Nanopore GridION device for 24 hours, with live basecalling using the high accuracy model, and the option require_two_barcodes. Quality control and filtering by expected amplicon size was performed using NanoPlot and NanoFilt (De Coster et al., 2018), followed by primer removal using cutadapt (Martin, 2011). The data was then polished once using Minimap2 and Racon (Li, 2018; Vaser et al., 2017). Demultiplexing, OTU clustering at 97% sequence similarity (min size = 2), chimera removal, and OTU table generation were performed using VSEARCH (Rognes et al., 2016). Taxonomy was assigned to the 16S rRNA and 18S rRNA gene amplicons using SILVA release 138.1 (Quast et al., 2013), and SINTAX as implemented in VSEARCH.

### Data analysis

Data visualization and statistical analyses were conducted in R (https://www.r-project.org/) using RStudio (https://www.rstudio.com/). Alpha and beta diversity metrics were computed with the ampvis2 package (v.2.8.3). The Kruskal–Wallis test followed by Dunn’s post hoc test with Bonferroni correction and the Mann–Whitney *U* test were applied to assess, respectively, significant spatial and temporal differences in physicochemical parameters, PFAS concentrations, and diversity indices. Differences in microbial community structure across spatial and temporal gradients were evaluated by using Analysis of Similarities (ANOSIM). Principal component analysis (PCA) was applied to standardized environmental variables using the vegan package (v.2.6–4) aiming to identify the main gradients structuring spatiotemporal variability.

Spearman’s rank correlations were calculated between the relative abundance of the top 30 most abundant taxa. To define this subset, the total number of sequence reads for each taxon across all samples were aggregated and the 30 taxa with the highest overall counts at the genus level were selected. Results were visualized as clustered heatmaps using the pheatmap package (v.1.0.12). Similarly, the taxonomic composition bar plots (Fig. 6) were constructed using the top 20 most abundant orders.

Furthermore, Redundancy Analysis (RDA) was performed using the *vegan* package to quantify the proportion of variation in microbial community composition explained by the combined influence of physicochemical parameters and PFAS concentrations. The significance of the global RDA model and the explanatory power of individual environmental vectors were determined via permutation tests (*999* permutations). Boxplots were generated using ggplot2 (v.3.4.4) to represent the distribution of physicochemical parameters and diversity indices across sites and seasons. A significance threshold of *p* < 0.05 was adopted for all tests. Custom R scripts were developed for ordination, statistical analyses, and plotting, with the aid of generative language models (ChatGPT, OpenAI, GPT-4o, 2024), and critically reviewed and adapted by the authors.

## Results and discussion

### Surface water quality

**Fig. 2:**
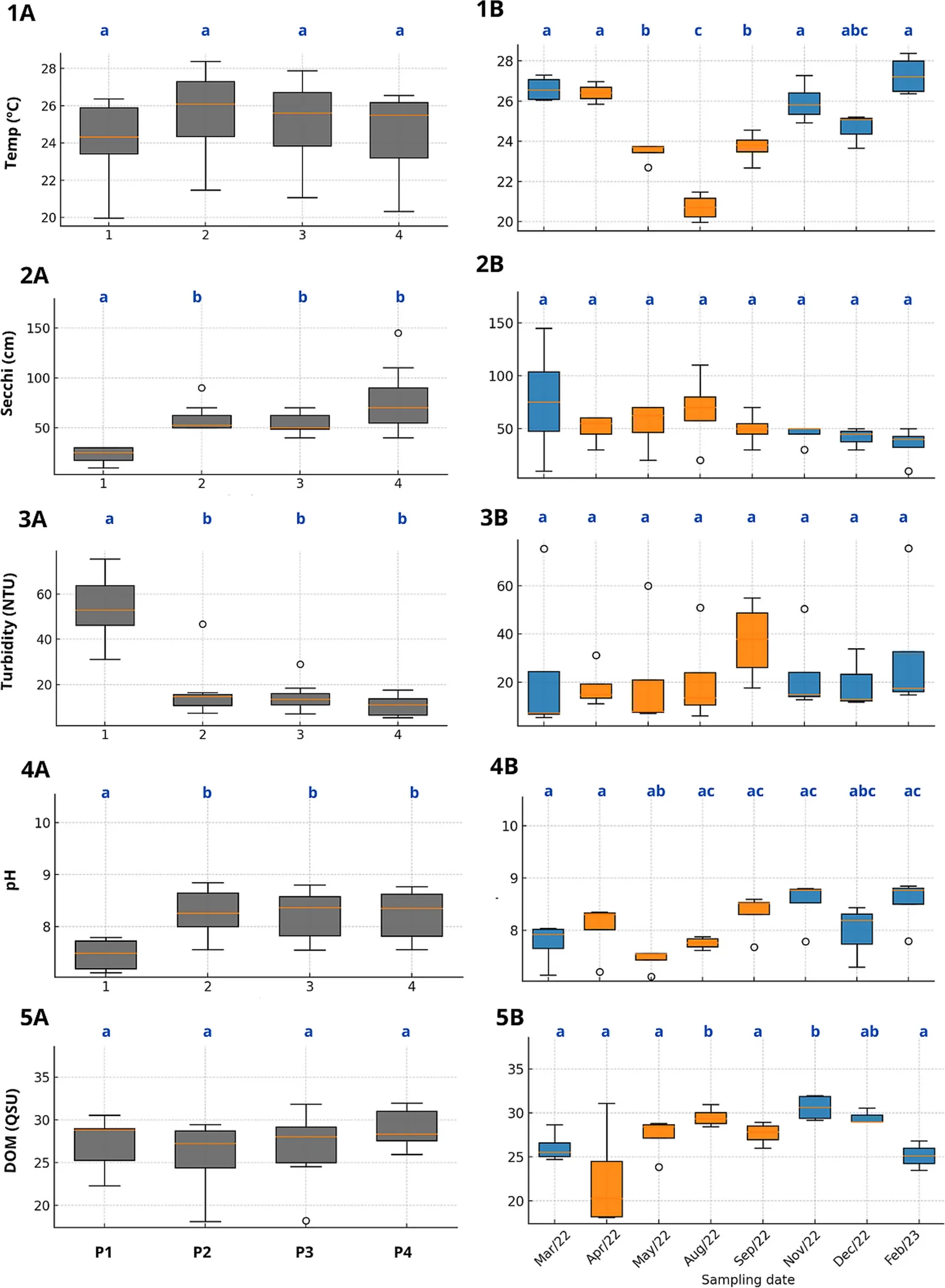

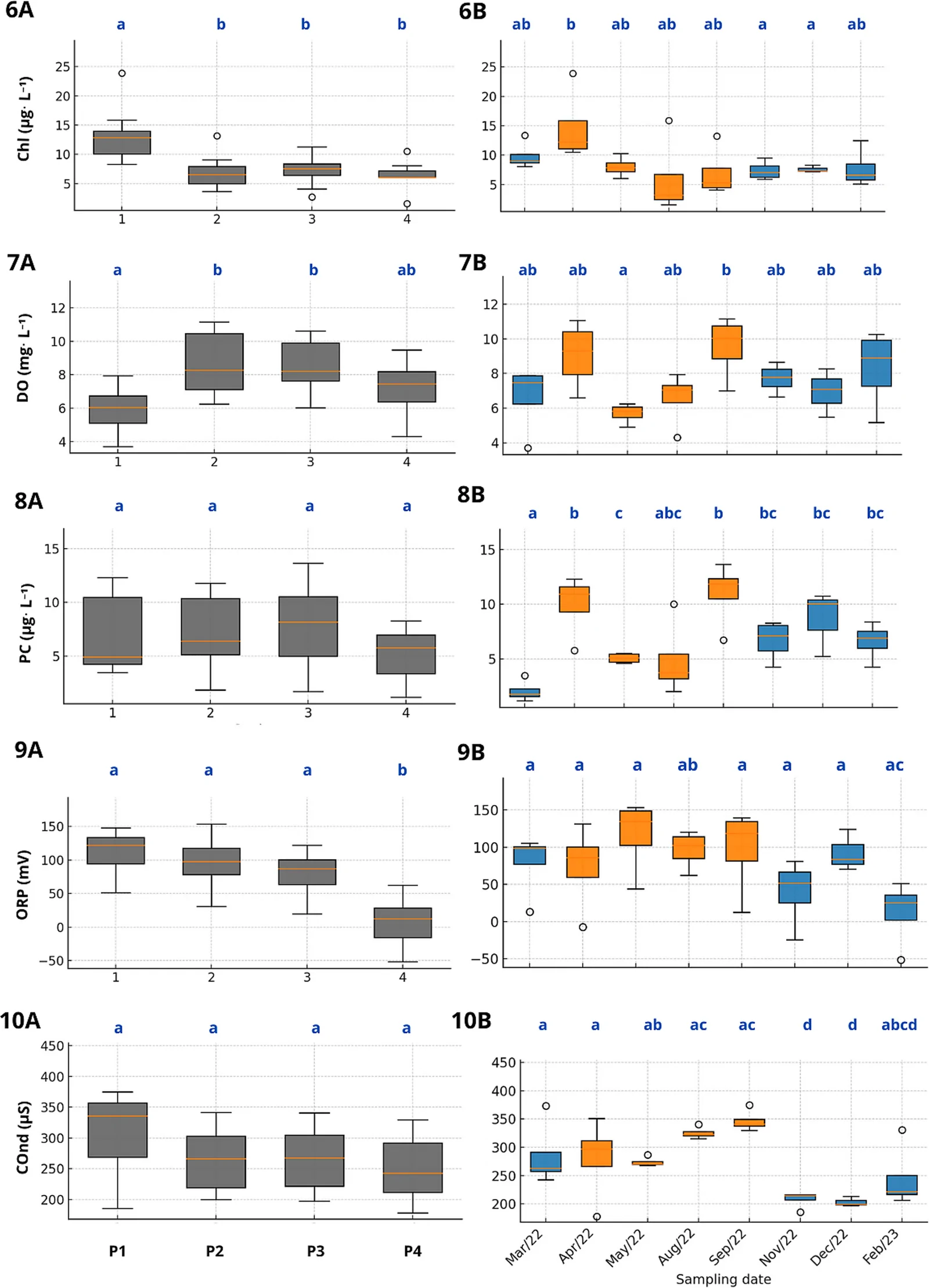
Boxplots representing the spatial (P1, P2, P3, P4) and temporal (orange = dry season, Apr–Sep; blue = rainy season, Oct–Mar) variations of physicochemical parameters measured in surface water in Pampulha Lake. Panels display: **1A** and **1B**—temperature (°C); **2A** and **2B**—transparency (Secchi disc depth, m); **3A** and **3B**—turbidity (NTU); **4A** and **4B**—pH; **5A** and **5B**—fluorescent dissolved organic matter (fDOM, QSU); **6A** and **6B**—chlorophyll-*a* (µg∙ L^−1^); **7A** and **7B**—dissolved oxygen (DO, mg∙L^−1^); **8A** and **8B**—total algal pigments (TAL PC, µg∙ L^−1^); **9A** and **9B**—oxidation–reduction potential (ORP, mV); and **10A** and **10B**—electrical conductivity (µS). Lowercase letters indicate statistically significant differences among groups

Surface water temperature in Pampulha Lake remained relatively stable across spatial gradients during the study period (Fig. 2(1A)). This pattern is consistent with the dynamics of shallow tropical reservoirs, where high solar irradiance throughout the year tends to homogenize the upper water column, limiting wind-driven mixing and strong thermal gradients (Figueredo et al., 2016). Despite this general stability, some fluctuations were observed: the highest temperatures occurred in April, possibly reflecting short-term climatic anomalies, while the lowest were recorded in August during austral winter (Fig. 2(1B)). Thus, both extremes occurred in the dry season.

Precipitation patterns showed greater variability than temperature, with marked peaks in November (260.8 mm), December (222.4 mm), and February (99.4 mm), and minimal rainfall in March (4.8 mm), April (4.8 mm), and August (0 mm), while May reached 23.4 mm. These rainfall pulses play a key role in modulating hydrological inputs, sediment resuspension, and nutrient loading, thereby indirectly influencing the biogeochemical processes of the reservoir (Sadro et al., 2011). The combination of relatively stable thermal conditions with variable rainfall highlights the importance of hydrological dynamics, rather than temperature, as the dominant driver of seasonal change in this tropical reservoir.

Marked spatial differences were observed for physicochemical conditions (Fig. 2(1A to 10 A)). Transparency by Secchi depth was significantly lower at P1 compared to the other sites (P2–P4), while turbidity was highest at P1, reflecting heavy influence of inflows enriched of untreated sewage and organic matter. pH also differed significantly between P1 and the other points, with more variable and often reduced values near inflows. Chlorophyll-*a* concentrations were highest at P1, in agreement with its eutrophic and nutrient-enriched status, whereas dissolved oxygen (DO) was consistently lower in this point compared to P2 and P3. In contrast, oxidation–reduction potential (ORP) values peaked at P4, the deepest region of the reservoir, suggesting more oxidizing conditions in surface waters where inflow influence from tributaries is limited. These results confirm that P1 is the most impacted site, receiving direct sewage from tributaries and catchment inputs, while P4 represents a more diluted, less impacted zone with better water circulation and oxidation potential. Such heterogeneity is well documented in tropical reservoirs subject to urban effluents (Araújo & Azevedo, 2011; Rocha et al., 2024).

Seasonal variation was also evident (Fig. 2B). Conductivity values were higher in March and May relative to November, August, and September, reflecting concentration of dissolved ions during the dry season due to reduced dilution and enhanced evaporation (e.g., May vs. November: *p* = 0.028; May vs. August: *p* = 0.028). Chlorophyll*-a* concentrations peaked in April, being significantly higher than in May (*p* = 0.028) and November (*p* = 0.028), thus indicating the occurrence of a phytoplankton bloom likely triggered by increased light availability and nutrient recycling in the early dry season. These blooms were accompanied by higher DO levels in April and September relative to May (April vs. May: *p* = 0.028; September vs. May: *p* = 0.028), consistent with increased photosynthetic oxygen production during periods of elevated algal biomass (Chen et al., 2018). Elevated DO also occurred simultaneously with increased pH in August, September, November, and February, reflecting carbon dioxide uptake during elevated photosynthesis rate (Zinabu, 2002). Conversely, ORP decreased significantly in February 2023 compared to August, indicating a shift towards more reduced conditions during the end of the rainy season. This likely resulted from enhanced organic matter input from runoff, stimulating microbial respiration and oxygen consumption (Wen et al., 2016). Taken together, these observations reveal the strong seasonal influence of hydrological dynamics on water quality in Pampulha Lake, with runoff-driven organic matter inputs and concentration processes shaping the biogeochemical environment during the rainy and dry seasons, respectively (Pinto Coelho 2002).

### PFAS occurrence and distribution

Seven of the 24 PFAS compounds analyzed were detected above the method quantification limits (MQL): PFBS and PFHxA (short-chain), and PFDA, PFOA, PFDoA, Br-PFOS, and n-PFOS (long-chain). Concentrations ranged from 1.9 µg∙L^−1^ (Br-PFOS) to 124.2 µg∙L^−1^ (PFBS) (Table supplementary material). These results reflect continuous contamination, most likely linked to untreated sewage inputs that have historically dominated Pampulha Lake’s catchment (Starling et al., 2024).

Both short- and long-chain PFAS are commonly reported in surface waters worldwide, yet the dominance of PFBS and PFOA in Pampulha Lake highlights their widespread use and environmental persistence (Podder et al., 2021). Although exploratory plots suggested spatial and temporal variation, statistical tests (Mann–Whitney and Kruskal–Wallis) did not detect significant differences among sampling points nor seasons (*p* > 0.05). Nevertheless, high PFAS levels measured in Pampulha Lake approached thresholds of ecotoxicological concern, raising questions about chronic exposure and long-term ecological risks.

**Fig. 3:**
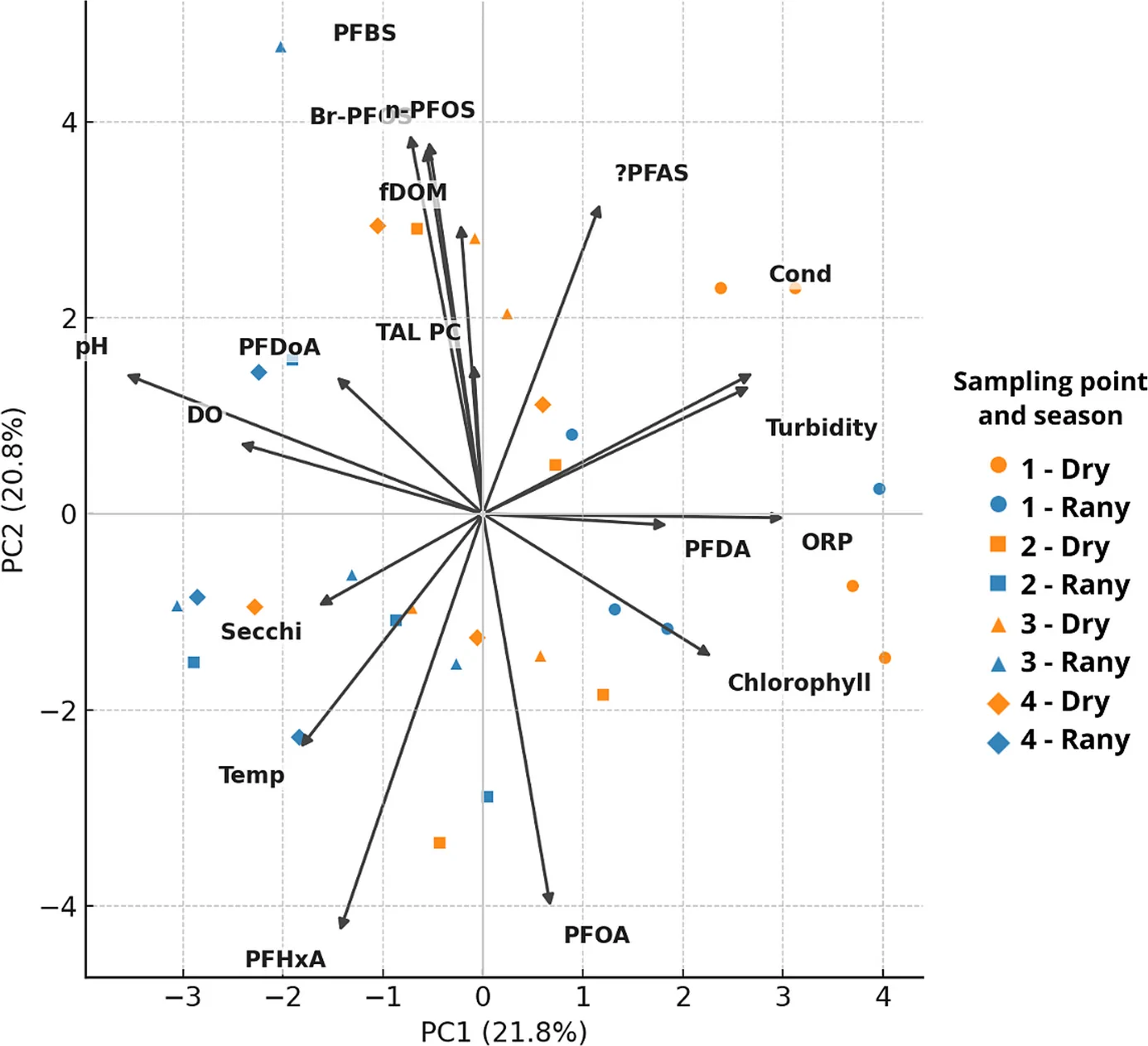
Principal component analysis (PCA) biplot showing the ordination of sampling points based on physicochemical variables in Pampulha Lake. The PCA was performed using standardized values of transparency (Secchi depth, m), temperature (°C), turbidity (NTU), pH, fDOM (QSU), chlorophyll-*a* (µg∙L^−1^), dissolved oxygen (DO, mg∙L^−1^), total algal pigments (TAL PC, µg∙L^−1^), oxidation–reduction potential (ORP, mV), electrical conductivity (µS∙cm^−1^), and PFAS (µg∙L^−1^). Arrows represent the loadings of environmental variables, indicating their direction and correlation strength with the principal components. Sampling points are grouped by spatial location (P1–P4) and/or temporal period (March 2022 to February 2023), highlighting patterns among observations

In contrast, the PCA integrating physicochemical and PFAS data revealed variations across both space and time dimensions (Fig. 3). Along PC1 (21.8% variance explained), samples from August, September, and November pulled apart from those of March, May, and February, suggesting strong seasonal effects driven by rainfall intensity, runoff, and associated turbidity. PC2 (20.8%) differentiated sites, particularly P1, reflecting local drivers such as restricted flow or the influence of infrastructure. Importantly, the PCA indicated a clear seasonal partitioning between short- and long-chain PFAS. Short-chain compounds (PFBS, PFHxA, PFOA) were primarily associated with rainy-season conditions, suggesting that their transport is strongly influenced by surface runoff and diffuse catchment sources (Ahrens, 2011; Guo et al., 2023). By contrast, long-chain PFAS (Br-PFOS, n-PFOS, PFDA, PFDoA) clustered with variables typical of the dry season (e.g., conductivity, pH, ORP), supporting the hypothesis that these congeners persist and accumulate under less diluted and more stable conditions (Ahrens & Bundschuh, 2014; Giesy & Kannan, 2001). Taken together, these results reinforce the conceptual framework distinguishing mobility of short-chain PFAS versus the persistence and bioaccumulative potential of long-chain congeners (Brunn et al., 2023; Wang et al., 2017). This dynamic is particularly critical in tropical urban reservoirs, where intense rainfall events enhance contaminant mobilization, while dry-season stratification favors retention and accumulation.

### Microbial diversity patterns

**Fig. 4:**
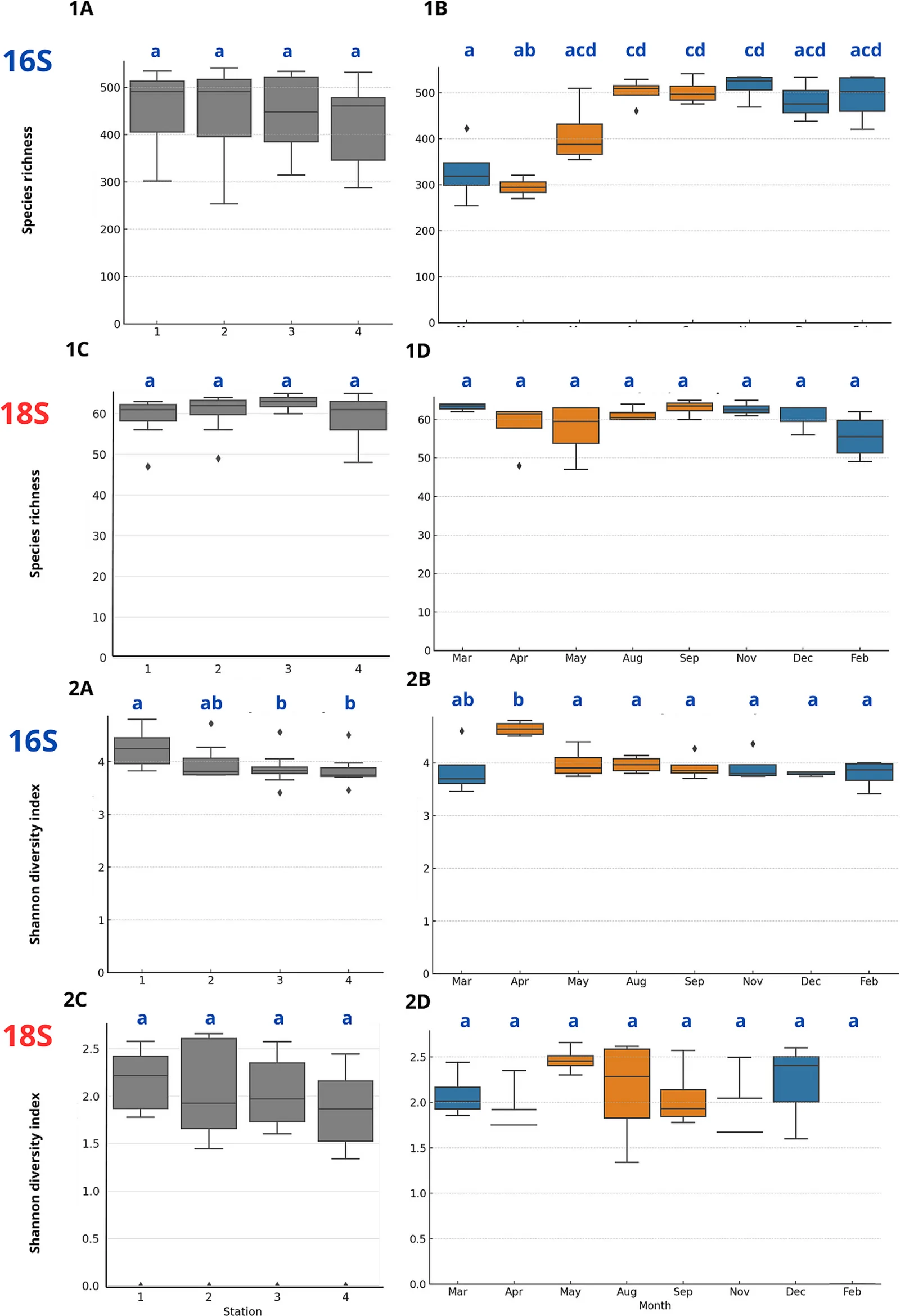
Species richness and Shannon diversity across samples based on 16S rRNA- and 18S rRNA gene sequences. Panels **1A** and **1B** show species richness for 16S rRNA; panels **1C** and **1D** show species richness for 18S rRNA gene amplicons. Panels **2A** and **2B** display Shannon diversity indices for 16S rRNA gene amplicons; panels **2C** and **2D** display Shannon diversity for 18S rRNA gene amplicons. Lowercase letters above the boxplots indicate statistically significant differences between groups (*p* < 0.05)

Microbial diversity analyses revealed contrasting responses between bacterial and eukaryotic communities. Based on 16S rRNA gene amplicons, richness displayed significant temporal variation, whereas Shannon diversity showed weaker spatiotemporal trends (Fig. 4(A–B)). Richness differed significantly among sampling months (Kruskal–Wallis: *H* = 19.79, *p* = 0.006), with lower values observed in March and April, possibly reflecting environmental stress conditions or post-bloom dynamics. Such declines may be associated with shifts in nutrient availability or organic matter influx following rainfall events, processes known to reduce community complexity in eutrophic systems (Lindh et al., 2015). Post hoc Dunn tests indicated significantly lower richness in April compared with November (*p* = 0.049).

Shannon diversity tended to peak at site P1 and during April, indicating greater community evenness during a period of environmental transition, although global temporal variation was not statistically significant (Kruskal–Wallis: *H* = 11.89, *p* = 0.104). This pattern suggests that April may represent a transient ecological window characterized by intermediate disturbance or community recovery phases, possibly linked to the chlorophyll-*a* peak observed during the same period. Spatial variation in Shannon diversity approached significance (*H* = 7.73, *p* = 0.052), with higher average values observed at site P1.

By contrast, richness and Shannon diversity based on 18S rRNA gene data did not reveal consistent spatial or temporal patterns (Fig. 4(C–D)). These results indicate that bacterial communities were more responsive to environmental fluctuations and chemical stressors, while microeukaryotic assemblages exhibited greater ecological tolerance or dispersal capacity (Fuhrman et al., 2015; Logares et al., 2018).

The contrast between 16S rRNA and 18S rRNA gene datasets underscores the importance of using multiple molecular markers when assessing ecosystem responses. While 16S rRNA gene amplicons proved as highly sensitive in detecting short-term environmental and pollutant-driven shifts, the relative stability of 18S rRNA gene assemblages highlights the broader ecological tolerance of microeukaryotes in eutrophic tropical lakes.

### Community structure and ordination analyses

**Fig. 5:**
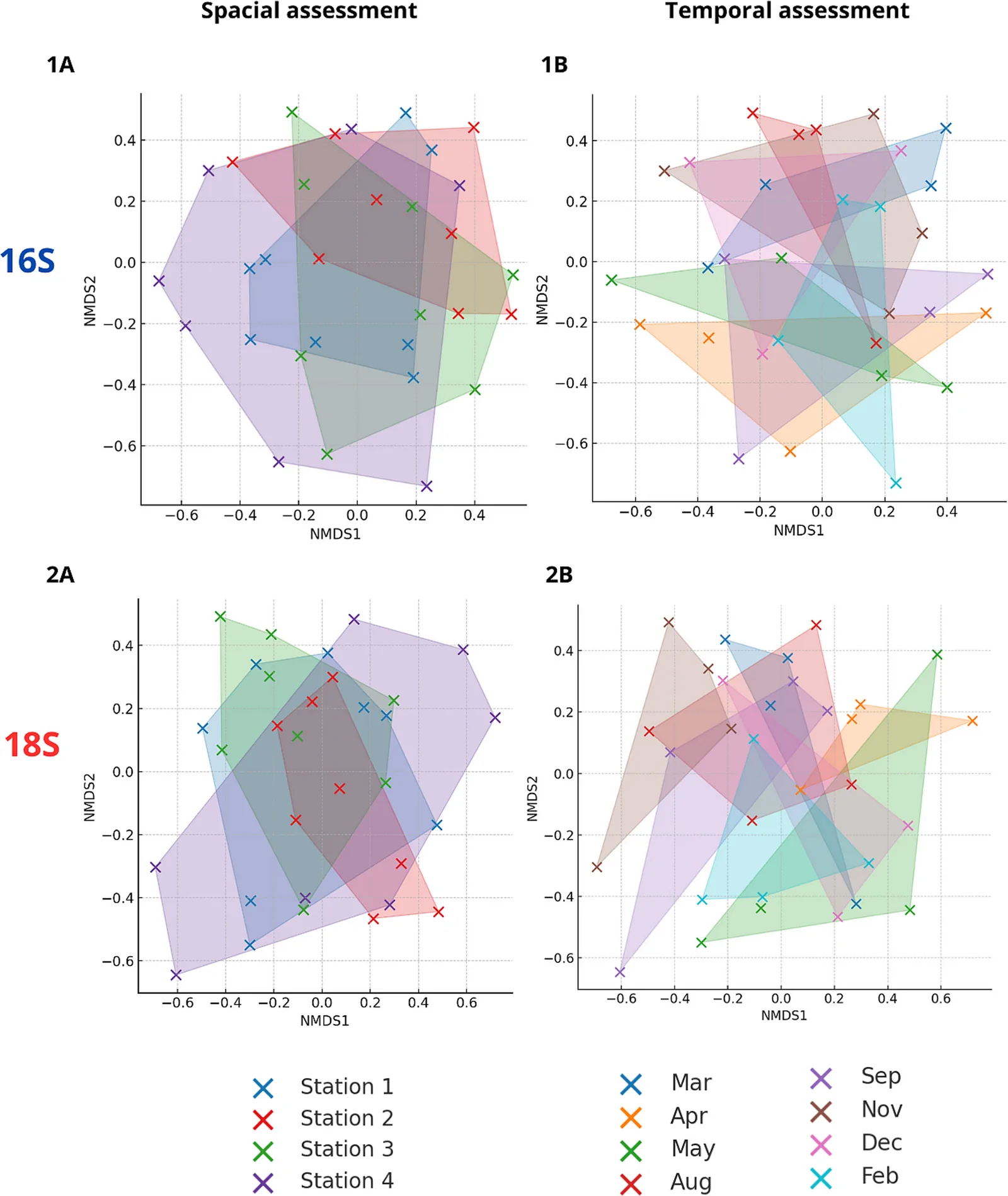
Non-metric multidimensional scaling (NMDS) ordination of microbial community structure based on Bray–Curtis dissimilarity for (1) prokaryotic communities (16S rRNA, stress value 18.7270) and (2) microeukaryotic communities (18S rRNA, stress value 17.6822). Each point represents a sampling unit, colored according to sampling stations (1–4), with convex hulls enclosing temporal replicates from the same station (**A**) and the shape area representing groups to the sampling month (March 2022 to February 2023) (**B**)

Non-metric multidimensional scaling (NMDS) analyses revealed clear differences in community structure between prokaryotes and microeukaryotes (Fig. 5). Prokaryotic communities based on 16S rRNA gene sequences showed a strong temporal structuring (ANOSIM, *R* = 0.449, *p* < 0.001), as samples clustered according to seasonal periods. This pattern indicates that bacterial assemblages are sensitive to hydrological and nutrient dynamics characteristic of tropical reservoirs, where rainfall-driven pulses strongly modulate microbial turnover.

Spatial differences were also evident, although less pronounced than temporal ones but still significant (ANOSIM: *R* = 0.100, *p* = 0.034). In particular, site P1 consistently clustered separately from the other stations in the 16S rRNA gene amplicon dataset, reflecting the influence of untreated sewage inflows and operational variability of the adjacent water treatment facility. This site-specific separation supports the interpretation that localized pollution inputs can override broader seasonal signals, at least for bacterial assemblages.

By contrast, eukaryotic communities based on 18S rRNA gene sequences did not exhibit strong temporal or spatial clustering in the NMDS ordination (temporal structuring ANOSIM: *R* = 0.032, *p* = 0.295; and spatial structuring ANOSIM: *R* = 0.023, *p* = 0.289). The absence of distinct grouping suggests that microeukaryotic assemblages in Pampulha Lake may be shaped by broader ecological tolerances, higher dispersal potential, or greater resilience to short-term environmental variation. This stability contrasts with the more dynamic bacterial communities and aligns with previous studies in tropical and temperate aquatic systems showing that eukaryotic plankton may be buffered against rapid environmental fluctuations (Boëchat et al., 2014).

Taken together, NMDS results highlight a dual pattern: bacterial communities exhibit strong seasonal and site-specific responses, while eukaryotic assemblages appear comparatively stable. Such contrasting dynamics emphasize the value of integrating both markers (16S rRNA and 18S rRNA gene) to capture complementary aspects of microbial community structure in eutrophic tropical reservoirs.

### Taxonomic composition and putative ecological roles

**Fig. 6:**
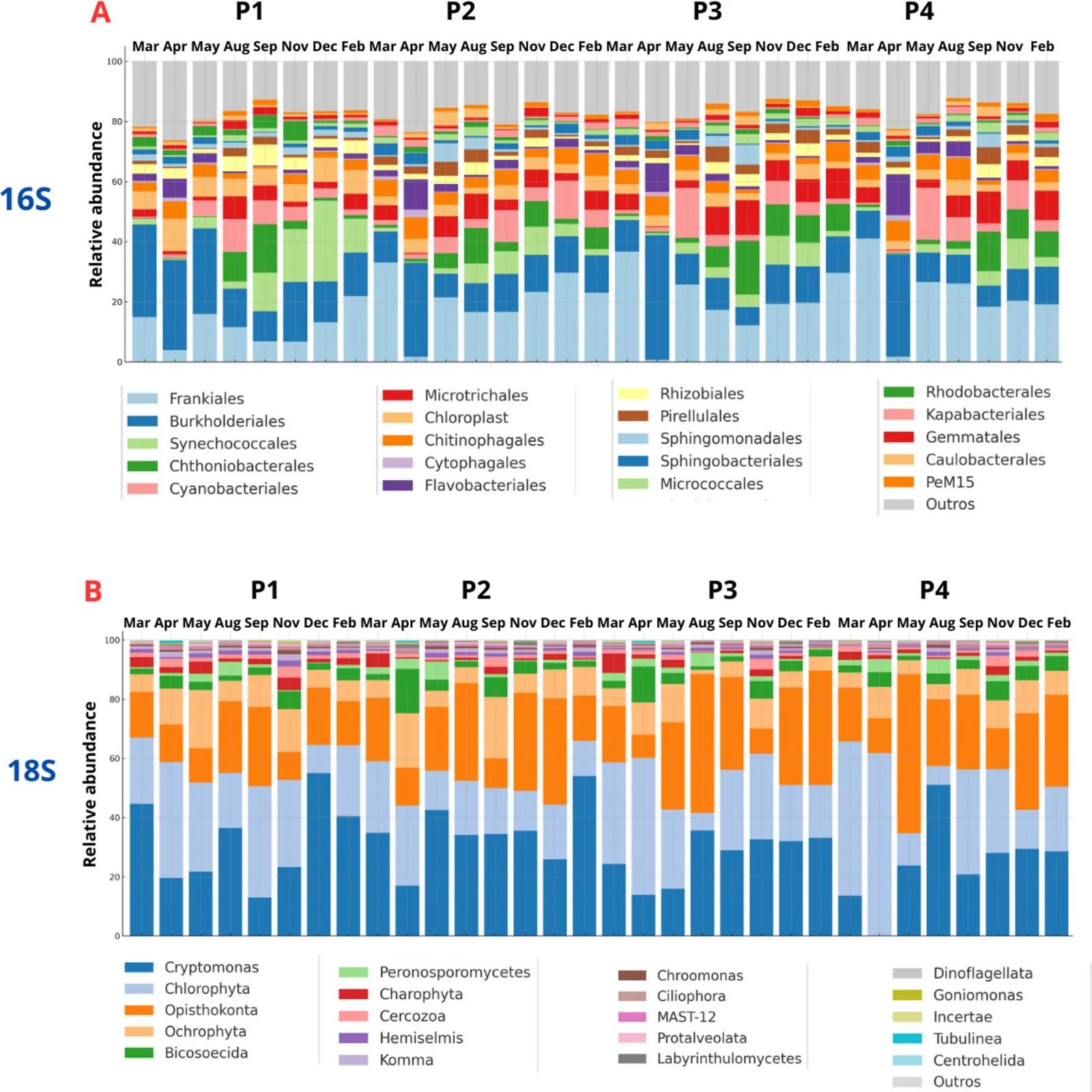
Relative abundance of orders detected via eDNA sequencing of 16S rRNA (**A**) and 18S rRNA (**B**) gene amplicons for each sampling point and campaign

The bacterial community based on 16S rRNA gene profiles was dominated by taxa typically associated with eutrophic and disturbed environments (Fig. 6A). Orders such as Frankiales, Burkholderiales, Synechococcales, Chthoniobacterales, and Cyanobacteriales were consistently abundant. Their dominance reflects high nutrient availability and organic matter inputs, which favor both autotrophic and heterotrophic lineages.

Synechococcales and Cyanobacteriales, known for oxygenic photosynthesis and harmful algal bloom formation, were aligned with elevated chlorophyll-*a* levels and are typical of nutrient-rich waters. This agrees with previous studies reporting cyanobacterial dominance in Pampulha Lake and other tropical urban reservoirs (Barçante et al., 2020; Paerl & Paul, 2012). Frankiales and Chthoniobacterales are often associated with soils and sediments, indicating particulate organic matter input and benthic–pelagic coupling. Burkholderiales, known for their metabolic versatility, contribute with nitrogen fixation and degradation of complex organic compounds (Poole et al., 2018).

Other taxa with known ecological roles included Chitinophagales, Cytophagales, and Flavobacteriales, which degrade algal exudates and labile dissolved organic carbon, processes that may be stimulated by sewage and bloom-derived organic matter (Newton et al., 2011). Pirellulales and Gemmatales, specialized in recalcitrant matter degradation and phosphorus turnover, were also detected, suggesting active nutrient recycling within the reservoir (Buckley et al., 2006; Dedysh & Ivanova, 2019). Taxa affiliated with Rhodobacterales, Microtrichales, and Rhizobiales have previously been associated with sulfur, nitrogen, and carbon cycling in aquatic ecosystems (Wagner-Döbler & Biebl, 2006; Poole et al., 2018). Although direct functional inference is beyond the scope of 16S rRNA gene metabarcoding, the presence of these groups may suggest potential ecological relevance in nutrient transformation and organic matter processing under eutrophic conditions.

Several groups, including Sphingomonadales and Sphingobacteriales, are well known for their ability to tolerate or degrade micropollutants and xenobiotics. Their detection in Pampulha Lake is consistent with the expected influence of complex effluent mixtures in urban reservoirs lacking proper sanitation infrastructure (Yu et al., 2020), which are common in low- and middle-income contexts. The presence of these bacteria in the study area highlights the selective pressure exerted by pollutants such as PFAS, pesticides, and other organic contaminants, as they may promote the enrichment of stress-tolerant lineages.

Furthermore, biofilm-associated bacteria (e.g., Caulobacterales, Sphingobacteriales) suggest the presence of redox-stratified microhabitats, supporting syntrophic interactions between cyanobacteria, heterotrophs, and mixotrophic algae. Biofilms create microscale redox gradients that allow for the coexistence of aerobic, microaerophilic, and anaerobic processes, favoring community resilience under fluctuating stress conditions (Flemming & Wuertz, 2019; Stewart & Franklin, 2008).

The eukaryotic community (18S rRNA gene) was dominated by chlorophyte algae (e.g., *Acutodesmus*, *Scenedesmus*, *Chlorella*, *Monoraphidium*, *Micractinium*, *Pseudopediastrum*), taxa commonly associated with eutrophic systems worldwide (Becker & Marin, 2009; Guiry, 2012). Their prevalence in Pampulha Lake is in alignment with the high chlorophyll-*a* levels observed. Mixotrophic taxa, including Cryptomonadales and *Chlamydomonas*, were also detected, reflecting ecological flexibility under variable light and nutrient conditions (Flynn et al., 2013). Heterotrophic protists such as *Cephalomonas*, *Obazoa*, and *Rhizaria* played a role in bacterial predation and nutrient regeneration (Sherr & Sherr, 2002). Occasional amplification of vascular plant DNA (Magnoliophyta) likely reflects runoff inputs or amplification of non-target DNA from sediment-rich samples.

The integration of the taxonomic composition and putative ecological roles of bacteria and eukaryotes reveal a highly diverse and ecologically resilient assemblage shaped by eutrophication and anthropogenic pressures in Pampulha Lake. Interactions between primary producers, degraders, and stress-tolerant taxa underpin functional redundancy, contributing to the persistence of ecosystem functioning even under intense chemical and nutrient stress.

### Correlations with PFAS and environmental gradients

**Fig. 7:**
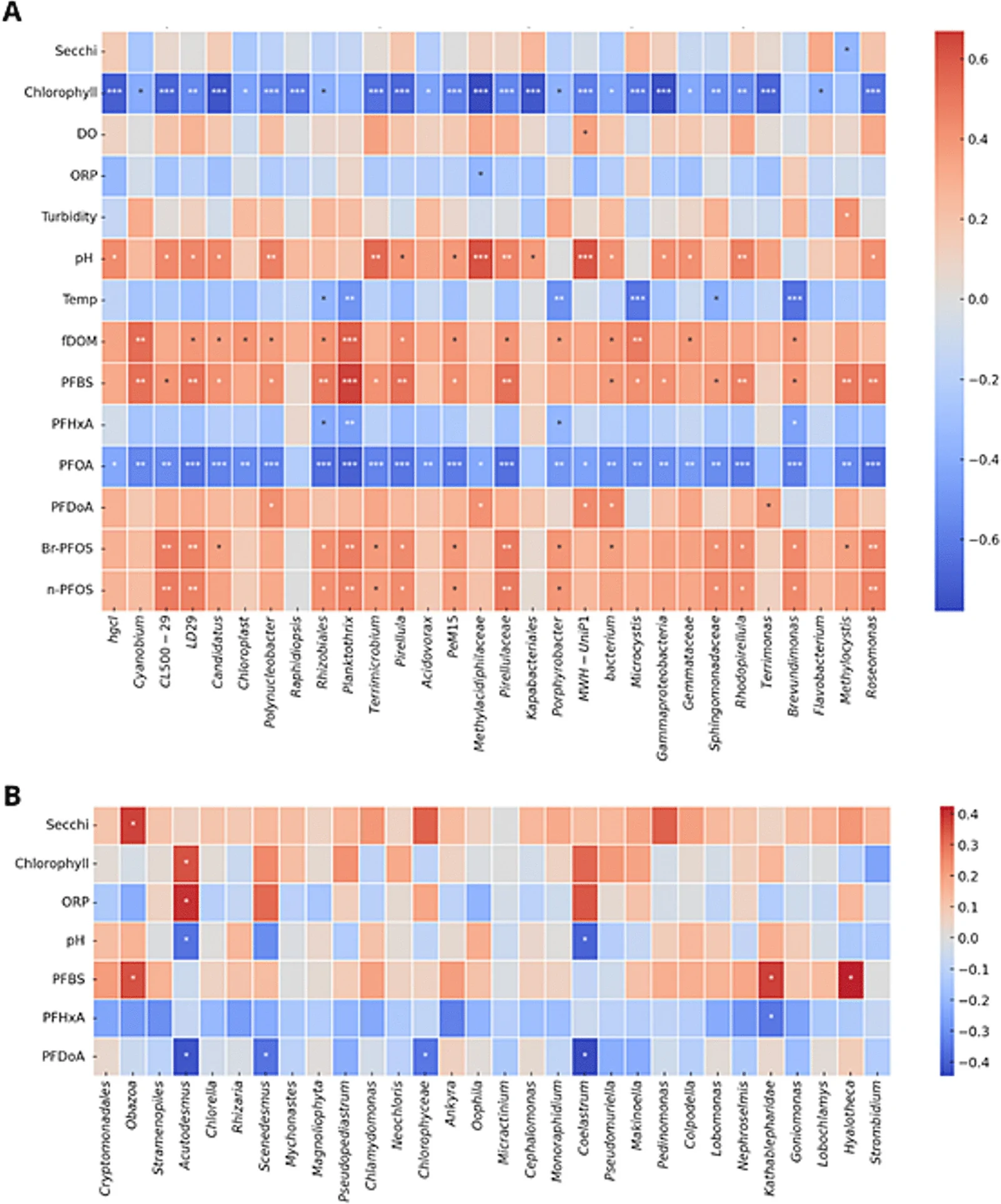
Heatmaps showing Spearman correlations between environmental variables and the 30 most abundant microbial taxa detected through 16S- (**A**) and 18S rRNA gene (**B**) sequencing. Each cell represents the correlation coefficient between the relative abundance of a taxon (columns) and an environmental variable (rows). Color intensity reflects the strength and direction of the correlation (red for positive, blue for negative), centered at zero. Statistical significance is indicated by asterisks: *p < 0.05, **p < 0.01, and ***p < 0.001

Spearman’s rank correlations between PFAS concentrations and microbial taxa revealed complex associations, highlighting the selective influence of these contaminants on community structure (Fig. 7).

Overall, negative correlations were more frequent with long-chain compounds, particularly PFOA. This pattern is consistent with known toxic effects of long-chain PFAS, including oxidative stress, disruption of cell membranes, and interference with energy metabolism (Laine et al., 2022; Liu et al., 2016). In contrast, positive correlations with sulfonated PFAS (PFBS, PFOS) were observed and appeared to favor certain bacterial taxa such as *LD29*, *CL500-29*, and *Rhodopirellula*. These groups may possess metabolic traits or structural adaptations enabling tolerance or potential biotransformation of PFAS, consistent with their ecological roles in stress resilience.

Cyanobacteria exhibited variable responses. Genera such as *Planktothrix* and *Raphidiopsis* correlated positively with PFOS, suggesting possible facilitation of bloom-forming species under PFAS exposure (Liao et al., 2025). By contrast, green algae (e.g., *Acutodesmus*, *Scenedesmus*, *Chlorella*) were negatively correlated with long-chain PFAS, reflecting their higher sensitivity to membrane disruption and oxidative stress. These results align with experimental studies reporting inhibition of algal growth and photosynthetic activity in the presence of PFAS, particularly long-chain congeners (Mojiri et al., 2023).

Heterotrophic bacterial taxa also showed differentiated responses. While some groups (e.g., *Roseomonas*, *Terrimicrobium*) correlated positively with sulfonated PFAS, suggesting selective enrichment under chemical stress, others (e.g., *Polynucleobacter*, *Actinobacteria* lineages) were negatively associated, reflecting possible vulnerability to PFAS toxicity.

Altogether, these correlations suggest that PFAS chain length and functional group (carboxylates vs sulfonates) are differentially shaped in concordance with the microbial community composition. Short-chain sulfonates appear to allow selective persistence or even proliferation of tolerant taxa, while long-chain compounds exert inhibitory effects on both prokaryotic and eukaryotic lineages. These contrasting responses identify potential bioindicators of PFAS contamination, particularly lineages such as LD29, CL500-29, and *Rhodopirellula*, which consistently correlate with contaminant gradients.

### Multivariate correlation (RDA)

**Fig. 8:**
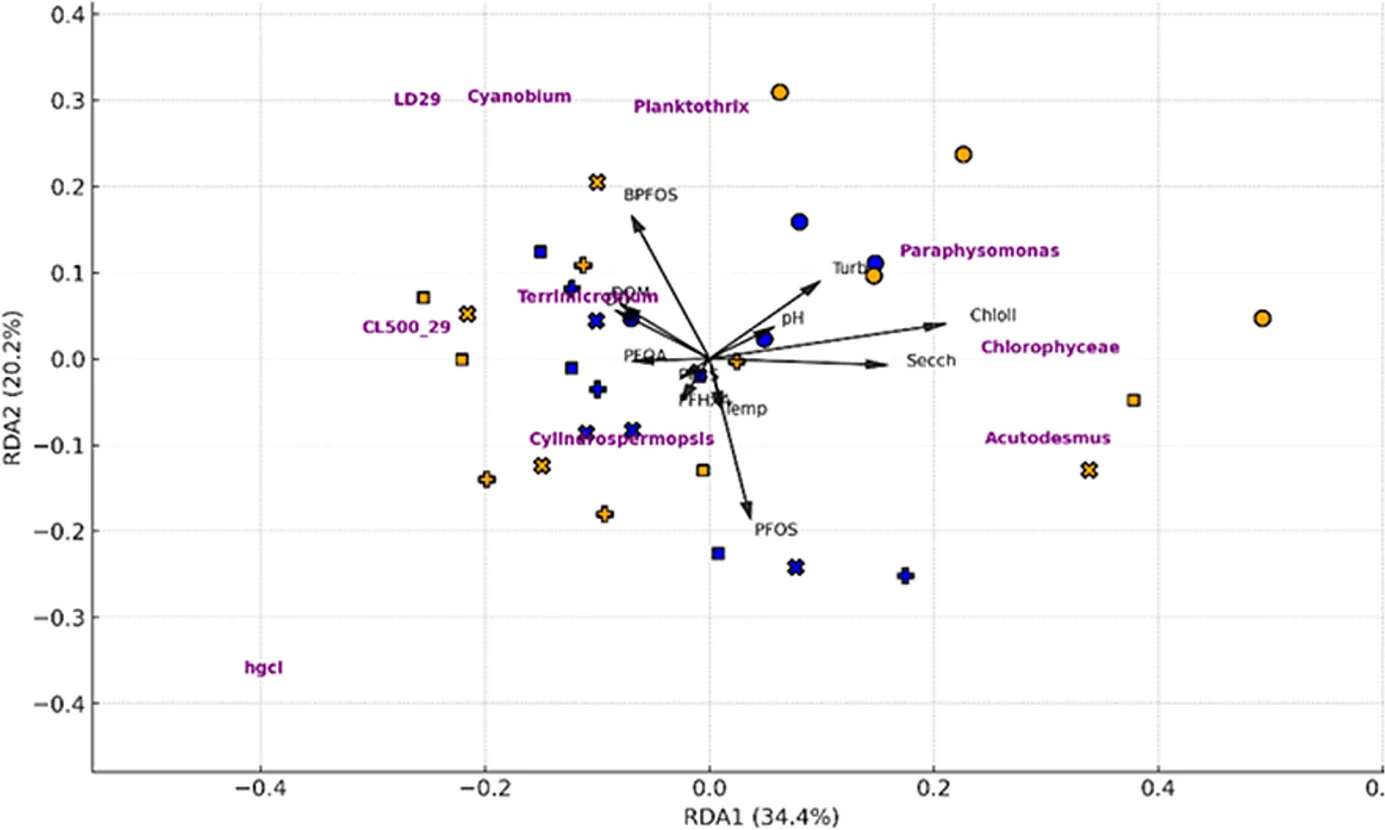
Redundancy analysis (RDA) ordination combining microbial community composition from 16S- and 18S rRNA gene datasets. The first two canonical axes explain 34.4% (RDA1) and 20.2% (RDA2) of the total constrained variation, respectively. Sampling periods are represented by color: blue for dry season and yellow for rainy season. Sampling sites are distinguished by symbols: P1—filled circle (●); P2—cross (×); P3—filled square (■); P4—plus sign (+). Environmental vectors indicate the direction and strength of correlations between environmental variables and community structure. Only variables with significant correlation to the ordination axes, determined by permutation tests (*p* < 0.05), are shown

Redundancy analysis (RDA) revealed that a substantial proportion of the variation in microbial community composition could be explained by the combined influence of physicochemical parameters and PFAS concentrations (Fig. 8). The first two canonical axes accounted for 54.6% of the total variance, indicating strong coupling between environmental gradients and microbial assemblages.

Autotrophic and mixotrophic taxa, including *Acutodesmus*, *Scenedesmus*, and *Planktothrix*, were positively associated with variables such as higher transparency, elevated pH, and PFOS concentrations. This association suggests that phototrophic lineages may be simultaneously stimulated by favorable light and nutrient recycling conditions, while also tolerating sulfonated PFAS. Such patterns are consistent with studies showing that PFOS can accumulate in phototrophs and interact with cell membranes without immediately eliminating growth (Wu et al., 2022).

In contrast, heterotrophic bacterial taxa, including *Cyanobium*, *Terrimicrobium*, and members of *Verrucomicrobia*, were correlated with elevated dissolved organic matter (DOM) and a mixture of PFAS compounds, particularly long-chain carboxylates (PFDA, PFDoA, PFOA). This suggests that these groups may exploit PFAS-driven alterations in organic matter quality or availability, possibly reflecting both indirect and direct selective pressures.

Notably, toxin-producing cyanobacteria such as *Raphidiopsis* showed a positive association with PFOS and pH. This raises concern about potential synergistic effects between PFAS exposure and harmful algal bloom development in tropical urban reservoirs. Such findings expand previous observations linking eutrophication, pH shifts, and cyanobacterial proliferation (Paerl & Paul, 2012; Figueredo et al., 2016).

Overall, the RDA demonstrates that microbial community structure is shaped by a combination of environmental conditions and PFAS chemistry. Whereas sulfonated PFAS aligned with autotrophic and bloom-forming taxa, long-chain carboxylates were more strongly linked to heterotrophic and DOM-associated lineages. This differentiation highlights compound-specific ecological niches and provides a mechanistic basis for the contrasting patterns observed in the correlation and diversity analyses presented herein.

### Ecological implications

The integration of physicochemical, PFAS, and eDNA data provides a comprehensive view of how persistent contaminants and eutrophication jointly shape microbial communities in tropical urban reservoirs influenced by lack of sanitation infrastructure (Shittu et al., 2023), a fairly common context in low- and middle-income countries. Results from Pampulha Lake demonstrate that PFAS act as additional selective pressure factors, superimposed on established hostile stress conditions caused by untreated sewage input, high nutrient loads, and organic matter accumulation. Similar findings have been recently documented in various freshwater systems, where PFAS not only act as direct toxicants but also interact with nutrient-driven processes and restructure planktonic microbiomes (Wu et al., 2022). For instance, studies on riverine ecosystems (Guo et al., 2023; Li et al., 2024) have demonstrated that PFAS emissions significantly alter microbial community networks, confirming that these persistent contaminants act as a ubiquitous selective pressure across different freshwater environments, exacerbating the ecological stress caused by urbanization and untreated effluents (Dominique et al., 2025).

Results also revealed that bacterial assemblages were more sensitive to environmental and chemical gradients than eukaryotic communities. The consistent correlations between long-chain PFAS and decreased bacterial richness and diversity reinforce the assumption that these compounds disrupt microbial homeostasis through oxidative stress and membrane interference (Laine et al., 2022; Liu et al., 2016). These toxicological mechanisms and inhibitory effects have been similarly observed across broader microbial assemblages, significantly impacting the growth and photosynthetic activity of green microalgae and complex microbial communities exposed to varying PFAS concentrations (Davis et al., 2024; Mojiri et al., 2023). In contrast, positive associations between sulfonated PFAS and specific lineages (e.g., LD29, CL500-29, *Rhodopirellula*, *Terrimicrobium*) highlight the possibility of selective enrichment of tolerant or opportunistic taxa. Bioremediation potential of cyanobacteria is underscored by studies which demonstrate that *Synechocystis* sp. PCC 6803 can tolerate and partially sequester PFAS contaminants like PFOA and PFOS at concentrations up to 1 mg·L^−1^ (Marchetto et al., 2021). Such groups could serve as potential molecular bioindicators of PFAS exposure in eutrophic systems, in line with the growing recognition of eDNA-based monitoring tools for detecting ecological stressors (Deiner et al., 2017; Pawlowski et al., 2018).

Microeukaryotic assemblages appeared comparatively stable, suggesting broader ecological tolerance or higher dispersal potential. Nevertheless, their composition was still influenced by eutrophication, with dominance of chlorophytes and mixotrophs typical of nutrient-rich waters. This observation echoes patterns reported in other tropical lakes, where phytoplankton communities show resilience despite fluctuating chemical stressors (Harke et al., 2016). By contrasting 16S rRNA and 18S rRNA gene amplicon results, this study underscores the complementary information provided by different molecular markers in anthropogenically impacted surface water: bacteria reveal rapid, sensitive responses to stressors, whereas eukaryotes reflect longer-term structural stability.

Beyond specific taxonomic responses, the combined evidence suggests that PFAS contamination may exacerbate ecological risks already associated with eutrophication. The observed association between PFOS and toxin-producing cyanobacteria (e.g., *Raphidiopsis*) is particularly concerning, as it implies a possible synergistic interaction between chemical pollution and harmful algal bloom development. This hypothesis is strongly supported by recent metabolomic analyses demonstrating that PFOS and other PFAS can shift cyanobacterial metabolic pathways in eutrophic waters, actively facilitating bloom formation (Liao et al., 2025). Furthermore, PFAS are known to regulate community coalescence and food web interactions in planktonic microbiomes (Wu et al., 2022), suggesting that chemical pollution does not merely coexist with eutrophication, yet it can fundamentally modify the competitive dynamics of harmful cyanobacteria, as highlighted in global syntheses of bloom dynamics under multiple stressors (Paerl & Paul, 2012; Harke et al., 2016). These interactions could compromise water quality, ecosystem services, and even human health in urban areas relying on freshwater systems.

Altogether, these findings emphasize the need to include PFAS monitoring in ecological risk assessments and management strategies for tropical urban reservoirs. Integrating eDNA metabarcoding with chemical and physicochemical data provides a powerful approach for identifying bioindicators, guiding conservation actions, and informing water quality policies. This integration reflects broader calls to incorporate emerging contaminants into environmental monitoring frameworks and water governance strategies (Ahrens & Bundschuh, 2014; Allan et al., 2021).

## Conclusions

Collectively, findings obtained herein demonstrate that microbial community structure in urban tropical freshwater systems is shaped by the combined influence of environmental gradients and persistent pollutants such as PFAS. Negative correlations with long-chain compounds contrast with positive responses to sulfonated congeners, reflecting compound-specific microbial interactions. Associations with pH, temperature, DOM, and chlorophyll further highlight the importance of trophic dynamics and climate-linked variability in shaping microbial assemblages and ecosystem function.

Several bacterial taxa, including LD29, CL500-29, *Rhodopirellula*, and *Terrimicrobium*, consistently responded to multiple stressors and thus represent promising candidates for molecular bioindicators. These results emphasize the dual role of microbial communities as sensitive to environmental disturbance and as practical tools for integrated biomonitoring in eutrophic and pollution-impacted freshwater systems. The combined use of 16S-rRNA and 18S rRNA gene datasets proved essential for capturing both bacterial and eukaryotic responses, underscoring the value of multi-marker approaches in ecological assessment.

This study provides new insights into PFAS-driven ecological processes in tropical urban reservoirs and demonstrates the utility of eDNA metabarcoding for ecological risk assessment. Expanding this framework to other tropical freshwater systems and integrating functional genomic approaches (e.g., metagenomics, metabolomics) is critical to advance understanding of microbial adaptation, resilience, and their role as sentinels of anthropogenic stress.

## Supplementary information

Below is the link to the electronic supplementary material.

ESM 1 (XLSX 9.58 KB)

## Data Availability

The raw 16S and 18S rRNA gene sequencing data produced during this study have been deposited at the European Nucleotide Archive (ENA) under accession number PRJEB113636. Other datasets generated and/or analyzed during this study are available from the corresponding author upon reasonable request.

## Declarations

### Use of generative AI and AI-assisted technologies in the writing process

During the preparation of this work, the author(s) used ChatGPT (OpenAI, GPT-4o, 2024) to assist in drafting custom R scripts for data visualization and ordination analyses, as well as to improve the language and readability of the manuscript. After using this tool, the author(s) thoroughly reviewed, verified, and edited all outputs, and take full responsibility for the content of the publication.
